# The chloroplast genome sequence of *Michelia alba* (Magnoliaceae), an ornamental tree species

**DOI:** 10.1080/23802359.2016.1275850

**Published:** 2017-01-05

**Authors:** Damien Daniel Hinsinger, Joeri Sergej Strijk

**Affiliations:** a Plant Ecophysiology & Evolution Group, Guangxi Key Laboratory of Forest Ecology and Conservation, College of Forestry, DaXueDongLu 100, Nanning, Guangxi, China;; b State Key Laboratory for Conservation and Utilization of Subtropical Agro-bioresources, Guangxi University, Nanning, Guangxi, PR China

**Keywords:** Magnoliaceae, complete chloroplast genome, *Michelia odorata*, ornamental tropical trees

## Abstract

Magnoliaceae are both economically and ornamentally important trees. Despite extensive studies in this family, the taxonomy of *Michelia* L. remains unclear, as well as the taxonomical status of *Michelia alba*. Herein, we report the complete chloroplast genome of *M. alba* DC. The chloroplast genome was 159,789 bp in length, with a large single-copy (LSC) region of 87,951 bp and a small single-copy (SSC) region of 18,798 bp, separated by two inverted repeat (IRs) regions of 26,570 bp. It contained 156 genes, including 83 coding genes, 68 tRNA genes, and 8 rRNA genes. The overall GC content was 39.3%, and 43.2%, 38.0%, 34.3%, in the IRs, LSC, and SSC regions, respectively. A phylogenetic analysis showed that *M. alba* is closely related to *M. odora*, with the genus *Michelia* nested inside *Magnolia*.

The Magnoliaceae comprise ca. 220–240 species (Nie et al. 2008), widely used for ornamental urban planting all around the world, especially the tropical and subtropical species for their fragrant flowers. *Michelia* L. (≈30 species) is similar to *Magnolia* L., except that flowers are clustered among leaves and not terminal as in *Magnolia*, and recent molecular data suggested that the former could be a synonym of the later (Nie et al. 2008). *Michelia alba* D.C. is an evergreen tall tree native to Indonesia and now widespread in tropical and subtropical countries (Nooteboom 1985) for it fragrant flowers. Because wild or fruiting trees are unknown, *M. alba* is commonly considered as a hybrid between *M. champaca* and *M. montana* (Nooteboom 1985). However, no molecular study has investigated the taxonomical status of *M. alba* (but see Nie et al. 2008, where *M. alba* was closely related to *M. champaca*). Here, we report the complete chloroplast sequence of *M. alba* to provide resources for delineation of the taxonomical status of this species and its potential horticultural varieties.

We extracted genomic DNA from fresh *M. alba* leaves collected from the Guangxi University (GXU) campus (22°50'20.5”N, 108°17'23.1”E Nanning, China – voucher HINSINGER_2016-NNG1 and deposited in the herbarium of the College of Forestry of Guangxi University, Nanning, China), using a modified SDS protocol (Hinsinger & Strijk 2015; Cvetkovic et al. 2016). Library construction and sequencing were performed by Novogene (Beijing, China), according to the Illumina HiSeq2500 system manufacturer instructions. We performed a *de novo* assembly of the chloroplast genome with org.asm v0.2.05 (http://pythonhosted.org/ORG.asm/) followed by manual adjustments and a iterative mapping step (see Hinsinger and Strijk, 2016) to correct for potential assembly mistakes. We used Geneious R9 v9.1.6 (Biomatters Ltd, Auckland, New Zealand) to transfer the annotations from the congeneric *M. odora* sequence (NC023239).

The complete cp genome of *M. alba* (GenBank accession KY204085) was 159,789 bp in length, with a 87,951 bp large single-copy (LSC) region and a 18,798 bp small single-copy (SSC) region, separated by 2 26,570 bp inverted repeat (IRs). It contained 156 genes, including 83 CDS, 68 tRNA genes, and 8 rRNA genes. The overall GC content was 39.3%, and 43.2%, 38.0%, 34.3% in the IRs, LSC, and SSC, respectively.

We used RaxML as implemented in Geneious R9 v9.1.6 to construct a Maximum-Likelihood phylogenetic tree including 10 available representative species in Magnoliaceae (Figure 1). As expected, *M. alba* was closely related to *M. odora*, forming a clade included in *Magnolia*. Bootstrap values were high for all nodes but the relationship between *M. yunnanensis* and the ((*M. kobus*, *M. liliifera*), *M. acuminata*) clade. Interestingly, this grouping was already poorly supported in Nie et al. (2008), suggesting the lack of informative sites was not the cause of this low value. Finally, the placement of *M. liliifera* as the sister taxa of *M. kobus* suggested a mistaken labelling for *M. liliiflora*, as this species was closely related to *M. kobus* in Nie et al. (2008).

**Figure 1.**
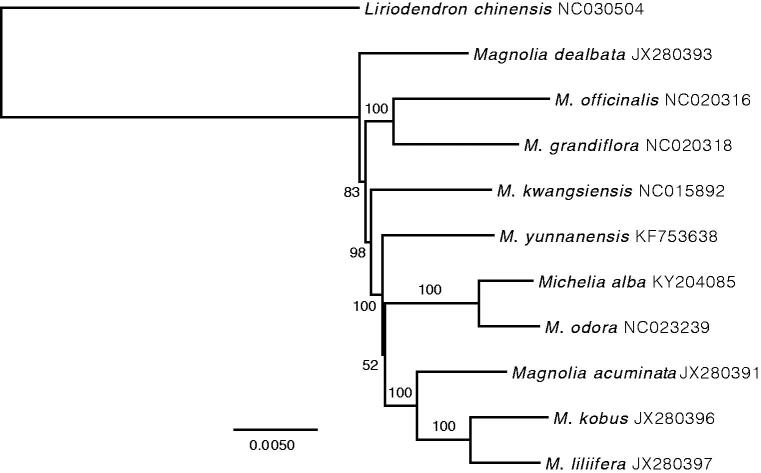
ML phylogenetic tree of 10 selected Magnoliaceae chloroplast sequences, plus the chloroplast sequence of *M. alba*. The tree is rooted with *Liriodendron chinensis*. Bootstraps (1000 replicates) are shown at the nodes. Scale in substitution per site.

We expect this sequence will help to clarify the hybrid status of *M. alba*, and provide additional genomic resources for Magnoliaceae studies.
